# A Mini-Review on the Effect of Docosahexaenoic Acid (DHA) on Cerulein-Induced and Hypertriglyceridemic Acute Pancreatitis

**DOI:** 10.3390/ijms18112239

**Published:** 2017-10-25

**Authors:** Yoo Kyung Jeong, Hyeyoung Kim

**Affiliations:** Department of Food and Nutrition, Brian Korea 21 PLUS Project, College of Human Ecology, Yonsei University, Seoul 03722, Korea; yookyung60@yonsei.ac.kr

**Keywords:** acute pancreatitis, cerulein, docosahexaenoic acid, hyperlipidemia

## Abstract

Acute pancreatitis refers to the sudden inflammation of the pancreas. It is associated with premature activation and release of digestive enzymes into the pancreatic interstitium and systemic circulation, resulting in pancreatic tissue autodigestion and multiple organ dysfunction, as well as with increased cytokine production, ultimately leading to deleterious local and systemic effects. Although mechanisms involved in pathogenesis of acute pancreatitis have not been completely elucidated, oxidative stress is regarded as a major risk factor. In human acute pancreatitis, lipid peroxide levels in pancreatic tissues increase. Docosahexaenoic acid (DHA), an omega-3 polyunsaturated fatty acid (C22:6n-3), exerts anti-inflammatory and antioxidant effects on various cells. Previous studies have shown that DHA activates peroxisome proliferator-activated receptor-γ and induces catalase, which inhibits oxidative stress-mediated inflammatory signaling required for cytokine expression in experimental acute pancreatitis using cerulein. Cerulein, a cholecystokinin analog, induces intra-acinar activation of trypsinogen in the pancreas, which results in human acute pancreatitis-like symptoms. Therefore, DHA supplementation may be beneficial for preventing or inhibiting acute pancreatitis development. Since DHA reduces serum triglyceride levels, addition of DHA to lipid-lowering drugs like statins has been investigated to reduce hypertriglyceridemic acute pancreatitis. However, high DHA concentrations increase cytosolic Ca^2+^, which activates protein kinase C and may induce hyperlipidemic acute pancreatitis. In this review, effect of DHA on cerulein-induced and hypertriglyceridemic acute pancreatitis has been discussed. The relation of high concentration of DHA to hyperlipidemic acute pancreatitis has been included.

## 1. Introduction

Acute pancreatitis is an inflammatory disease of the pancreas, which may result in multiple organ dysfunction and increased cytokine release [1,2]. About 20–30% patients develop severe forms of this disease, involving local and systemic complications. The mortality rate of acute pancreatitis patients has decreased over the last decade due to improvements in critical care; however, the worldwide incidence of acute pancreatitis is still high [3].

There have been various experimental acute pancreatitis models including ischemia-reperfusion, retrograde administration of sodium taurocholate into the pancreatic duct, and cerulein-induced edematous pancreatitis models [4]. Cerulein-induced pancreatitis is the most well-characterized and widely used experimental model for acute edematous pancreatitis. Supramaximal stimulation of the pancreas with cerulein, a cholecystokinin (CCK) analog, induces intra-acinar activation of trypsinogen in rat pancreas [5]. Moreover, cerulein doses, higher than those that cause maximum pancreatic secretion of amylase and lipase [6,7], dysregulate the production and secretion of digestive enzymes, resulting in increasing their levels in serum, along with causing cytoplasmic vacuolization, death of acinar cells, edema formation, and infiltration of inflammatory cells into the pancreas [8,9]. Ederle et al. [10] reported that the mean CCK level in pancreatitis patients (260.3 ± 300.8 pg/mL) was significantly higher than that in the control subjects (56.6 ± 61.7 pg/mL). Shirohara and Otsuki demonstrated that plasma CCK levels increased during acute pancreatitis, including gallstone pancreatitis, since edema of the bile duct causes transient disturbances in bile flow into the duodenum. They suggested the usefulness of CCK receptor antagonists for the treatment of acute pancreatitis [11].

Ghrelin (GHRL) is an endogenous ligand for the growth hormone secretagogue receptor (GHS-R). A recent study showed that cerulein inhibited GHS-R and GHRL expression in the rat pancreatic acinar cells. GHRL stimulates its own expression and expression of its receptor in isolated pancreatic acinar cells and AR42J cells on the positive feedback pathway. This mechanism may explain the pancreatoprotective effect of GHRL in the process of acute pancreatitis [12]. Obestatin, 23-amino-acid-peptide, colocalized with GHRL in human pancreas, decreased serum level of proinflammatory IL-1β and improved pancreatic blood flow in rats with cerulein-induced acute pancreatitis [13] as well as ischemia/reperfusion-induced acute pancreatitis of rats [14]. These studies suggest that decreased GHRL may contribute to the development of acute pancreatitis.

Although the mechanisms involved in the pathogenesis of acute pancreatitis are not completely understood, oxidative stress is regarded as a major risk factor [15,16,17]. It has been reported that reactive oxygen species (ROS) are important mediators for the initiation and development of pancreatitis [18]. Cerulein-induced activation of nuclear factor-κB (NF-κB) and cytokine expression is potentially mediated by ROS that are produced by NADPH oxidase in the pancreatic acinar cells [19]. ROS generation induced by cerulein is mainly responsible for cytokine production in acinar cells via direct activation of inflammatory signaling, involving protein kinase C-δ (PKC-δ), NF-κB, activator protein-1 (AP-1), and janus kinase 2/signal transducer and activator of transcription 3 (JAK2/STAT3) [19,20,21].

Previously, we demonstrated that the omega-3 polyunsaturated fatty acids (PUFAs) may prevent oxidative stress-induced inflammation in the pancreas [22]. Studies involving human subjects also indicated that the use of enteral formula enriched with omega-3 PUFAs for the treatment of acute pancreatitis may be beneficial, evident by the shortened time for jejunal feeding and hospital stay [23]. Moreover, it also reduced the histological severity of acute pancreatitis [24,25,26]. Parenteral therapy with omega-3 PUFAs decreased histopathologic severity in acute necrotizing pancreatitis via early inhibition of prostaglandin synthesis and reduction of lipid peroxidation [27].

DHA shows antioxidant and anti-inflammatory effects on various cells and tissues. Therefore, the beneficial effects of DHA for prevention and treatment of various diseases have been extensively investigated [28]. Understanding the underlying mechanism responsible for the inhibition of acute pancreatitis by DHA could help in the identification of novel therapeutic treatment options, thereby preventing undesirable complications or fatal outcomes.

Hypertriglyceridemia is associated with acute pancreatitis. A range of 3–38% of patients with acute pancreatitis is related to hypertriglyceridemic pancreatitis [29,30,31,32,33,34]. Both primary (genetic) and secondary disorders of lipoprotein metabolism (e.g., uncontrolled diabetes, alcoholism, medications, and pregnancy) may be associated with hypertriglyceridemic pancreatitis [35]. Perfusion of the pancreas with fatty acid (FA) has induced pancreatic edema [36] and the activation of trypsinogen to initiate acute pancreatitis in mice [37]. Hypertriglyceridemia (HTG) contributes to and accelerates the inflammatory cascade and pancreatic tissue damage in rats [38]. Furthermore, large amounts of FA inhibited mitochondrial complexes I and V and decreased ATP levels in acinar cells, which induced mitochondrial toxicity in pancreatic acinar cells [39]. Ca^2+^ signals are necessary for normal acinar cell secretory function [40]. However, abnormal, prolonged elevation of cytosolic Ca^2+^ is a critical trigger of pancreatitis via PKC activation [40]. Therefore, high levels of triglyceride (TG), FA, and Ca^2+^ may trigger pancreatic inflammation.

Since DHA reduces serum TG levels, it may prevent acute pancreatitis associated with HTG and FFA. On the other hand, a high concentration of DHA induces Ca^2+^-mediated activation of protein kinase C (PKC) isoforms (PKC-α, PKC-δ, PKC-ε, and PKC-ζ) and zymogen activation in the pancreatic acinar cells, which may promote acute pancreatitis [41].

Here, we review the antioxidant and anti-inflammatory effects of DHA on cerulein-induced experimental acute pancreatitis. In addition, the controversy associated with the effect of DHA on hypertriglyceridemic and hyperlipidemic acute pancreatitis has also been discussed.

## 2. Oxidative Stress and Inflammatory Signaling in Cerulein-Induced Acute Pancreatitis

Oxidative stress is a major risk factor associated with the pathogenesis of human acute pancreatitis. It has been demonstrated that pancreatic oxidative stress occurs during early stages of ROS induction [42]. Once produced, ROS can act as molecular triggers for inducing pancreatitis. They may attack biological membranes directly and/or trigger the accumulation of neutrophils and aid in their adherence to the capillary wall [43,44]. Therefore, it is likely that ROS play a central role in pancreatic inflammation and the development of additional pancreatic complications. In human acute pancreatitis, lipid peroxide levels in the bile and pancreatic tissues increase, while antioxidant vitamins decrease [45]. During pancreatitis, the increase in ROS may be related to reduced levels and activities of antioxidant enzymes, including superoxide dismutase (SOD) and catalase [46]. Depletion of pancreatic glutathione (GSH) occurs in the early phases of acute pancreatitis [47] and influences disease severity in rat models [48]. The activities of several antioxidant enzymes, including glutathione peroxidase, SOD, and catalase, and levels of antioxidant vitamins decrease in patients with human acute pancreatitis [49,50]. Serum lipid peroxide levels increased in human pancreatitis patients [51,52].

Evidences suggest that proinflammatory cytokines, such as IL-1β, TNF-α, and IL-6, act as mediators of acute pancreatitis [53,54,55]. IL-6 is a proinflammatory cytokine associated with acute phase responses during inflammation [56]. Elevated levels of IL-6 have been observed in patients with acute pancreatitis and are determinants of disease severity [57].

TNF-α is produced in pancreatic acinar cells in experimental acute pancreatitis model. It is an activator of immune cells and regulates the synthesis of other pro-inflammatory cytokines [58]. Pretreatment with GHRL in rats with intact sensory nerves reduced the pancreatitis-induced increase in plasma concentration of TNF-α [59]. GHRL induced anti-inflammatory cytokine IL-4, which suppressed IL-1β expression in cerulein-induced acute pancreatitis [59].

IL-1β is produced as a pro-enzyme and requires proteolytic cleavage by IL-1 converting enzyme (ICE) or by neutrophil proteases to develop maximal activity. IL-1β and ICE are expressed at low levels in mouse pancreas, but increase rapidly on cerulein stimulation or on a choline deficient, ethionine-supplemented diet [60]. Therefore, inhibition of IL-1β expression may prevent the development of acute pancreatitis [61].

Cerulein induces high ROS production and activates oxidation-sensitive NF-κB, thereby inducing high cytokine expression in freshly isolated pancreatic acinar cells (without inflammatory cells) in vitro [62]. NF-κB is known to regulate the expression of inflammatory cytokines such as IL-1β, TNF-α, and IL-6, which induce the acute and edematous form of pancreatitis. PKC-δ was shown to activate NF-κB in a mouse model of cerulein-induced acute pancreatitis. Activation of PKC-δ is necessary for NF-κB activation, which is responsible for the pathogenesis of acute pancreatitis [20]. Therefore, reducing ROS may prevent ROS-mediated activation of inflammatory signaling including NF-κB and expression of inflammatory cytokines in pancreas.

## 3. Antioxidant and Anti-Inflammatory Effects of DHA on Cerulein-Induced Acute Pancreatitis

DHA, at 20 and 50 μM, reduced ROS levels, leading to the inhibition of the JAK 2/STAT3 pathway in cerulein-stimulated pancreatic acinar cells [63]. In response to oxidative stress, activation of the JAK2/STAT3 pathway induces the expression of inflammatory cytokines [64,65]. Moreover, in pancreatic AR42J cells treated with cerulein, DHA acts as an agonist of peroxisome proliferator-activated receptor-γ (PPAR-γ). PPAR-γ induces the expression of glutathione and thioredoxin antioxidant systems in the murine hippocampal HT22 cells [66]. PPAR-γ inactivates STAT3 by directly interacting with STAT3 in cerulein-stimulated pancreatic acinar cells [67]. Since STAT3 regulate cytokine expression, PPAR-γ induction by DHA may be beneficial for inhibiting inflammation in pancreas. DHA upregulates the expression of antioxidant enzymes, including catalase, glutathione peroxidase, and manganese superoxide dismutase (SOD2), in the murine hippocampal HT22 cells and in rats during post-natal development [66,68,69]. In the cerulein-stimulated AR42J cells, DHA induces catalase expression [63]. Recent studies reported that PPAR-γ-specific agonists upregulate the expression of copper-zinc superoxide dismutase (SOD1) in primary endothelial cells [70], and SOD2 and glutathione peroxidase in the skeletal muscle cells, heart, and neurons [71]. Therefore, DHA may induce the expression of SOD1, SOD2, catalase, and glutathione peroxidase in cerulein-stimulated AR42J cells. Since oxidative stress is involved in the pathogenesis of acute pancreatitis, DHA may prevent and/or inhibit the development of acute pancreatitis by suppressing the inflammatory signaling pathways, such as JAK 2/STAT 3, in the pancreatic tissues.

It has been reported that cerulein upregulates IL-6 by activating NADPH oxidase to produce excess ROS in the pancreatic acinar cells [72]. Moreover, cerulein induces the nuclear transcription factor, activator protein-1 (AP-1), which regulates inflammatory cytokine gene expression [73]. DHA inhibited AP-1 activation and mRNA expression of IL-1β and IL-6 in the pancreatic acinar AR42J cells stimulated with cerulein [22]. In cerulein-treated rats, pretreatment with DHA (intraperitoneal injection, 13 mg/kg body) suppressed pancreatic edema formation, and increased lipid peroxidation, myeloperoxidase activity, and NF-κB activation in pancreatic tissues. DHA inhibited PKC-δ activation and increased the expression of antioxidant enzyme SOD1 in pancreatic tissues of cerulein-treated rats [74]. These results suggested that DHA may be beneficial for preventing the development of pancreatitis by suppressing the activation of PKC-δ and NF-κB, and inhibiting the expression of inflammatory cytokines.

**Figure 1:**
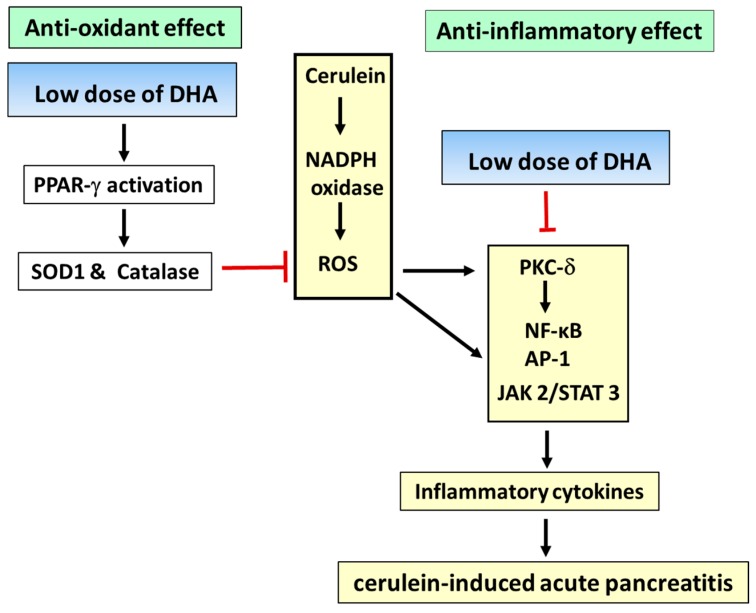
Proposed mechanisms of action of docosahexaenoic acid (DHA) in cerulein-induce acute pancreatitis. Cerulein induced the activation of NADPH oxidase, which produces large amounts of reactive oxygen species (ROS) in pancreatic acinar cells. ROS activate protein kinase C-δ (PKC-δ), which activates nuclear factor-κB (NF-κB). ROS also directly activate NF-κB, activator protein-1 (AP-1), janus kinase 2 (JAK2)/signal transducer and activator of transcription 3 (STAT3), and inflammatory cytokine expression in the cerulein-stimulated pancreatic acinar cells and rat models. The inflammatory events result in development of acute edematous pancreatitis. DHA induces activation of peroxisome proliferator-activated receptor-γ (PPAR-γ) and expression of the PPARγ-target gene, SOD1, and catalase. Since SOD1 and catalase scavenge ROS, DHA inhibits the ROS-mediated activation of inflammatory signaling (PKC-δ, NF-κB, AP-1, JAK2/STAT3) and inflammatory cytokine expression in cerulein-stimulated pancreatic acinar cells and animal models. Antioxidant and anti-inflammatory effects of DHA may be responsible for preventing the development of acute pancreatitis. The bars represent inhibition, while the arrows represent stimulation.

Taken together, DHA activates PPAR-γ and induces the expression of PPAR-γ-target gene, SOD1 and catalase, thereby inhibiting ROS-mediated activation of PKC-δ, NF-κB, AP-1, JAK2/STAT3, and inflammatory cytokine expression, in the in vitro cerulein-stimulated pancreatic acinar cells and in vivo rat models (Figure 1).

## 4. Hypertriglyceridemia and Acute Pancreatitis

Hypertriglyceridemia with serum TG levels ≥500 mg/dL (≥5.65 mmol/L), increases the risk of acute pancreatitis [75]. Therefore, lowering TG levels reduces the risk of pancreatitis. Both genetic and secondary disorders of lipoprotein metabolism are associated with hypertriglyceridemic pancreatitis. Pancreatic lipase-induced hydrolysis of TG and the subsequent formation of free fatty acids (FAs) trigger inflammation [35]. Earlier, Havel [76] proposed that, when high concentrations of FAs are present (concentrations exceed the binding capacity of plasma albumin), the FA molecules self-aggregate to form micellar structures with detergent-like properties. These FA micelles initially attack platelets and the vascular endothelium. Finally, they attack the acinar cells, thereby resulting in ischemia and pancreatic injury.

In the pathogenesis of acute pancreatitis, endothelial dysregulation, vascular leakage, and coagulation activation have been shown [77]. These pathologic events may be related to FA-induced vascular damage. Therefore, angiopoietin-2, which is associated with endothelial dysfunction, has been recently proposed as a marker of severity in acute pancreatitis [78]. Anticoagulant such as acenocoumarol has been used for treating experimental ischemia/reperfusion-induced acute pancreatitis in rats [79,80] and cerulein-induced pancreatitis rats [81].

## 5. Effect of DHA on Hypertriglyceridemic Acute Pancreatitis

**Figure 2:**
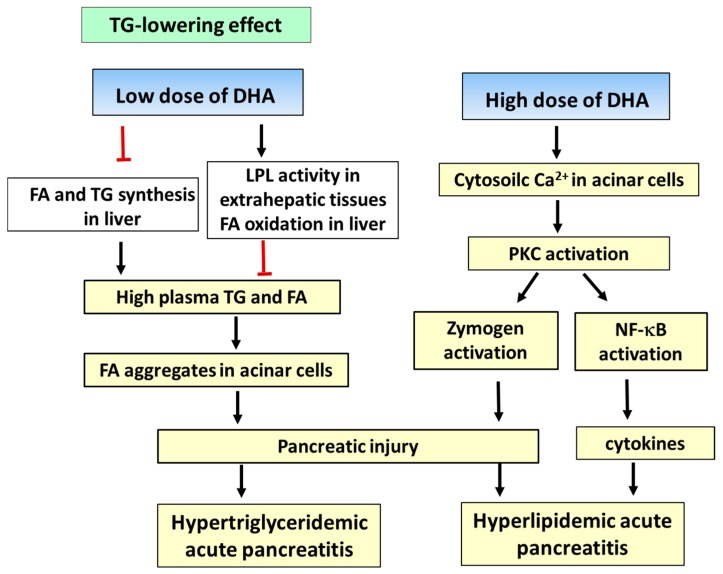
Proposed mechanisms of action of docosahexaenoic acid (DHA) in hypertriglyceridemic and hyperlipidemic acute pancreatitis. DHA inhibits triglyceride (TG) and fatty acid (FA) synthesis in the liver. DHA increases lipoprotein lipase (LPL) activity in the extrahepatic tissues and β-oxidation of FA in the liver and skeletal muscles, thereby contributing to the reduction of FA delivery to the liver and reducing plasma TG levels. High amounts of FAs induce pancreatic inflammation and injury. Therefore, the TG-lowering effect of DHA may prevent hypertriglyceridemic acute pancreatitis. On the other hand, a high concentration of DHA increases Ca^2+^ and activates PKC isoforms (PKC-α, PKC-δ, PKC-ε, and PKC-ζ) in pancreatic acinar cells, which may induce zymogene activation and pancreatic injury associated with hyperlipidemic acute pancreatitis. In addition, PKC activates NF-κB and induces inflammatory cytokine expression in pancreatic acinar cells. The bars represent inhibition, while the arrows represent stimulation.

Pharmacological interventions for lowering TG levels include statins, fibrates, nicotinic acid, and omega-3 PUFAs [82]. Omega-3 PUFAs reduce hepatic lipogenesis by inhibiting diacylglycerol acetyltransferase and phosphatidic acid phosphohydrolase, involved in TG synthesis, thereby decreasing TG production in the liver [83]. Omega-3 PUFAs inhibit hepatic FA synthesis by suppressing sterol regulatory element-binding protein (SREBP) 1c, a transcription factor that plays a key role in lipogenesis [84,85]. In addition, omega-3 PUFAs increase lipoprotein lipase activity in the extrahepatic tissues, including the adipose, heart, and skeletal muscles, and increase β-oxidation of FAs in the liver and skeletal muscles, thereby contributing to the reduction of FA delivery to the liver and reducing plasma TG levels [86]. Since FA aggregates induce pancreatic inflammation and injury, the TG-lowering effect of DHA may inhibit development of hypertriglyceridemic acute pancreatitis (Figure 2). At pharmacological doses (3–4 g/day), omega-3 PUFAs acids significantly reduce TG levels in hypertriglyceridemia patients [87]. Compared to EPA, DHA demonstrated a higher efficacy for the reduction of TG (DHA, −8 to −43.7%; EPA, +1.8 to −34.9%) [88,89]. Omega-3 PUFAs are effective in reducing the levels of TG and other lipids in hypertriglyceridemic patients treated with statins [90]. Currently, omega-3 PUFA-based formulations are being evaluated to ascertain whether the addition of omega-3 PUFAs to statin prevents the development of acute pancreatitis in patients with hypertriglyceridemia.

## 6. Effect of High Concentration of DHA on Hyperlipidemic Acute Pancreatitis

A high concentration (1 mM) of DHA induced a persistent increase in cytosolic Ca^2+^ concentration and upregulated the expression of PKC isoforms (PKC-α, PKC-δ, PKC-ε, and PKC-ζ) in mouse pancreatic acinar cells [41]. This increase in Ca^2+^ level and activation of PKC-α, PKC-δ, and PKC-ζ was similar to that observed using supramaximal concentrations of CCK in isolated pancreatic acinar cells [91]. Petersen and Sutton [92] reported that sustained elevation of Ca^2+^ concentration causes abnormal enzyme activation, vacuolization, and necrosis in acinar cells. In pancreatic acinar cells, PKC isoforms, namely, PKC-α, PKC-δ, PKC-ε, and PKC-ζ, have been identified [93]. PKC-δ incudes the premature activation of zymogen and NF-κB, and modulates the expression of inflammatory molecules in pancreatic acinar cells during experimental pancreatitis [94,95,96,97]. Therefore, high concentrations of DHA may promote the development of acute pancreatitis via activation of the PKC isoforms (Figure 2). Low concentrations of DHA (0.1 mM) had no effect of Ca^2+^ concentration in acinar cells [41]. These results support the proposed mechanism of pathogenesis of hyperlipidemic acute pancreatitis. Therefore, high DHA doses should be avoided for hyperlipidemia patients, for preventing the development of acute pancreatitis.

## 7. Other Omega-3 Fatty Acids and Pancreatitis

Cell membrane fatty acids are precursors of lipid mediators such as eicosanoids (prostaglandins: PG; thromboxanes: TX; leukotrienes: LT) [98]. Lipid mediators produced by DHA and eicosapentaenoic acid (EPA, C20:5n3) show anti-inflammatory properties [99]. EPA and DHA inhibit production of arachidonic acid-derived inflammatory eicosanoids [100]. Interestingly, the amounts of omega-3 PUFAs DHA, EPA, and docosapentaenoic acid (DPA, C22:5n3) decreased in the erythrocyte membrane phospholipids of acute pancreatitis patients compared to control subjects [101]. These studies suggest that reduction of omega-3 PUFAs may be related to the development of acute pancreatitis. In addition, the mixture of omega-3 PUFAs decreased Toll-like receptor 4, NF-κB p56, and inflammatory cytokine expression in the pancreas in the severe acute pancreatitis model of rats received retrograde infusion of sodium taurocholate into the pancreatic duct [102]. This study suggest that omega-3 PUFAs inhibit the TLR4/NF-κBp56 signaling pathway to suppress inflammatory cytokines in pancreas. Parenteral infusion of fish-oil-based lipid emulsion reduced systemic inflammatory cytokines and inflammatory eicosanoid levels in sodium taurocholate-induced acute pancreatitis [103].

Serum TG levels increase during pregnancy, which may elicit acute pancreatitis. Therefore, it is important to abrogate the rapid rise of TG levels in pregnancy [104]. Oral EPA prevented rapid increase in serum TG, suggesting that EPA administration may be a useful treatment for hypertriglyceridemic acute pancreatitis during pregnancy.

## 8. Conclusions and Future Directions

DHA treatment inhibited inflammatory mediators by reducing ROS generation and inflammatory cytokine expression in cerulein-induced experimental model for acute pancreatitis. DHA induces the expression of PPAR-γ-target gene, SOD1, and catalase, thereby inhibiting ROS-mediated activation of inflammatory signaling (PKC-δ, NF-κB, AP-1, JAK2/STAT3) and inflammatory cytokine expression in cerulein-stimulated pancreatic acinar cells and rat models. DHA-induced activation of PPAR-γ and catalase expression may be responsible for the anti-inflammatory effects of DHA in cerulein-induced acute pancreatitis. In addition, DHA inhibits FA and TG synthesis in liver. DHA activates LPL activity in extrahepatic tissues and FA oxidation in liver. Therefore, DHA reduces plasma levels of TG and FFA released from TG. Since FA aggregates induces pancreatic damage as well as vascular endothelial dysfunction, TG-lowering effect of DHA may inhibit hypertriglyceridemic acute pancreatitis. Combination of DHA and lipid-lowering drugs like statins may reduce the development of hypertriglyceridemic acute pancreatitis by decreasing TG levels. On the other hand, a high concentration of DHA may promote PKC-mediated inflammation, since PKC activates NF-κB and zymogen, which may cause hyperlipidemic acute pancreatitis. An understanding of how DHA interferes with inflammatory mediators is important for determining the effects of DHA when it is used in combination with traditional therapy for inhibiting inflammation in the pancreatic tissues.

## Abbreviations

AP-1	activator protein-1
CCK	cholecystokinin
DHA	docosahexaenoic acid
DPA	docosapentaenoic acid
EPA	eicosapentaenoic acid
FA	fatty acid
GSH	glutathione
GHRL	ghrelin
GHS-R	growth hormone secretagogue receptor
ICE	IL-1 converting enzyme
JAK	janus kinase
LPL	lipoprotein lipase
LT	leukotrienes
NF-κB	nuclear factor-κB
PG	prostaglandins
PKC	protein kinase C
PPAR-γ	peroxisome proliferator-activated receptor-γ
PUFAs	polyunsaturated acids
ROS	reactive oxygen species
SOD	superoxide dismutase
SREBP	sterol regulatory element-binding protein
STAT3	signal transducer and activator of transcription 3
TG	triglyceride
TX	thromboxanes
